# Most common robotic bariatric procedures: review and technical aspects

**DOI:** 10.1186/s13022-015-0019-9

**Published:** 2015-10-28

**Authors:** Pablo A. Acquafresca, Mariano Palermo, Tomasz Rogula, Guillermo E. Duza, Edgardo Serra

**Affiliations:** Division of Bariatric Surgery, CIEN-DIAGNOMED, Affiliated to the University of Buenos Aires, Av. Pte. Perón 10298 Ituzaingo, CP 1714 Buenos Aires, Argentina; Cleveland Clinic Foundation, Bariatric and Metabolic Institute, Cleveland, OH USA

**Keywords:** Bariatric surgery, Robotic surgery, Gastric by pass, Sleeve gastrectomy, Gastric band

## Abstract

Since its appear in the year 1997, when Drs. Cadiere and Himpens did the first robotic cholecystectomy in Brussels, not long after the first cholecystectomy, they performed the first robotic bariatric procedure. It is believed that robotically-assisted surgery’s most notable contributions are reflected in its ability to extend the benefits of minimally invasive surgery to procedures not routinely performed using minimal access techniques. We describe the 3 most common bariatric procedures done by robot. The main advantages of the robotic system applied to the gastric bypass appear to be better control of stoma size, avoidance of stapler costs, elimination of the potential for oropharyngeal and esophageal trauma, and a potential decrease in wound infection. While in the sleeve gastrectomy and adjustable gastric banding its utility is more debatable, giving a bigger advantage during surgery on patients with a very large BMI or revisional cases.

## Background

**Fig. 1:**
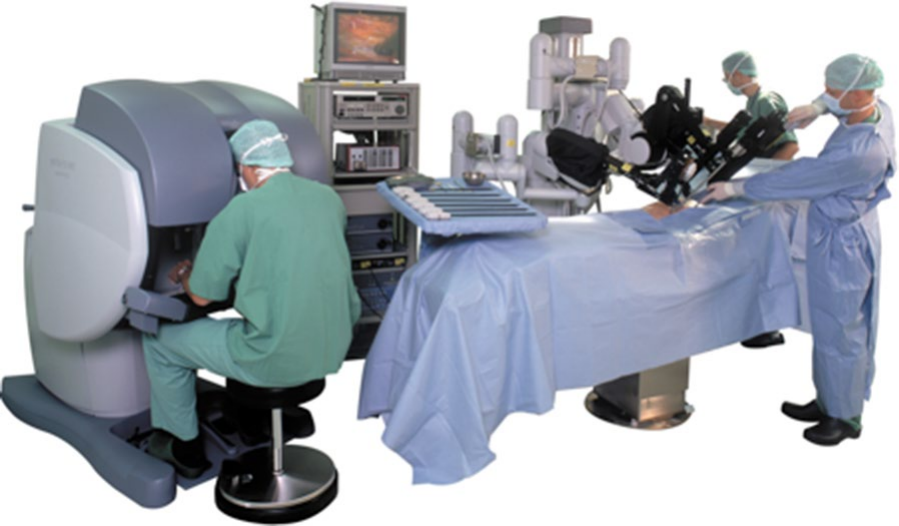
Da Vinci™ Robotic Surgical System from Intuitive Surgical, Inc., Sunny Vale, California

Since its appear in the year 1997, when Drs. Cadiere and Himpens did the first robotic cholecystectomy in Brussels [1], the da Vinci™ Robotic Surgical System from Intuitive Surgical, Inc., Sunny Vale, California (Fig. 1) has started a revolution in the surgery field. And of course, the bariatric surgery would not be excluded of this revolution. Not long after the first cholecystectomy, Dr. Cadiere and Himpens also performed the first robotic bariatric procedure. This was a robotic-assisted adjustable gastric banding, done to show the feasibility of the robotics platform [2]. Since then all procedures have been evolved into a robotic approach as an option to standard laparoscopy: adjustable gastric banding, sleeve gastrectomy, gastric bypass, biliopancreatic diversion with duodenal switch and revisional bariatric procedures. Nevertheless the current indications for using a robotic technique in bariatric surgery remain unclear.

With the digitization and robotization of laparoscopic procedures, the choice between conventional laparoscopy and robotic laparoscopy is now a controversial topic that concerns patients and surgeons alike.

To date, the robotic technique is reported to be at least as safe and effective as the conventional approach for several procedures, including hysterectomy, atrial ablation, cholecystectomy, nephrectomy, rectoplexy, fundoplication, and prostatectomy [3–9]. However, the specific benefits to both the patient and the surgeon are not yet well defined in most cases.

It is believed that robotically-assisted surgery’s most notable contributions are reflected in its ability to extend the benefits of minimally invasive surgery to procedures not routinely performed using minimal access techniques (i.e., total esophagectomies, coronary artery bypass grafting, and radical prostatectomies). And due to its characteristics may ultimately increase the number of physicians who are able to provide the benefits of minimal access surgery to their patients without the increased risks of complications associated with initial learning curves.

The additional advantages afforded by the use of minimally invasive surgical techniques, coupled with the desire to retain the natural ergonomics and visual advantages of open surgery, have propelled the development and progression of robot-assisted surgery which may allow surgeons to overcome many of the laparoscopy surgery difficulties: loss of depth perception, loss of natural hand eye co-ordination, loss of intuitive movement and loss of dexterity.

Depth perception is restored with a stereo visualization by using a two channel endoscope which sends both a left and right eye image back to the surgeon. The alignment of the surgeon’s hand motions to the surgical tool tip is both spatial and visual. To achieve spatial alignment, the system software aligns the motion of the tools with the camera frame of reference. To achieve visual alignment, the system projects the image of the surgical site atop the surgeon’s hands. Coupled together, spatial and visual alignment makes the surgeon feel as though his hands are inside the patient’s body [10].

The progress and development of these robotics characteristics will eventually provide all bariatric surgeons with the option of a minimally invasive approach.

## Adjustable gastric banding

Regarding its application to adjustable gastric banding, several publications have shown little benefit of the use of Robot for this procedure [11–13]. The largest series reported of robotic adjustable gastric banding (RAGB) included 287 patients and they were compared to 120 cases of standard laparoscopy (LABG). Outcomes were similar between the groups, with the exception of shorter operative times by 14 min in RAGB if the patient’s BMI was above 50 kg/m^2^ [14]. These data suggest that using the robot for LAGB does not alter the invasiveness to the patient, there are no clear benefits in terms of diminished adverse events. This may be the result of a ‘‘ceiling effect,’’ where the adverse events of conventional LAGB are already minimal, leaving very little room for improvement.

The utility of robotics for such a simple operation as we see, is matter of discussion. Maybe we can find its most useful application on revisional procedures of slipped bands and associated hiatal hernias or in patients with BMI above 50 kg/m^2^ were the robot has shown to short the operative time [13].

In cases of revisions of slipped bands associated with hiatal hernias usually these patients are converted to other procedures such as gastric bypass or sleeve gastrectomy, if they do not have severe reflux from their prior adjustable band. However, a subset of patients remains who want to keep their band. In this patients their hiatal hernia can be robotically repaired and the band positioned with hope of preventing recurrent adjustment difficulties and reflux.

In this small subset of patients, the stomach and band are fully exposed and a new retrogastric window is created superior to the prior band site. The hiatal crura is fully dissected and formally closed posteriorly and anteriorly over a 34 French gastric tube. The band is positioned into the new higher retrogastric window and sewn into place with gastrogastric anterior sutures.

### Surgical technique

**Fig. 2:**
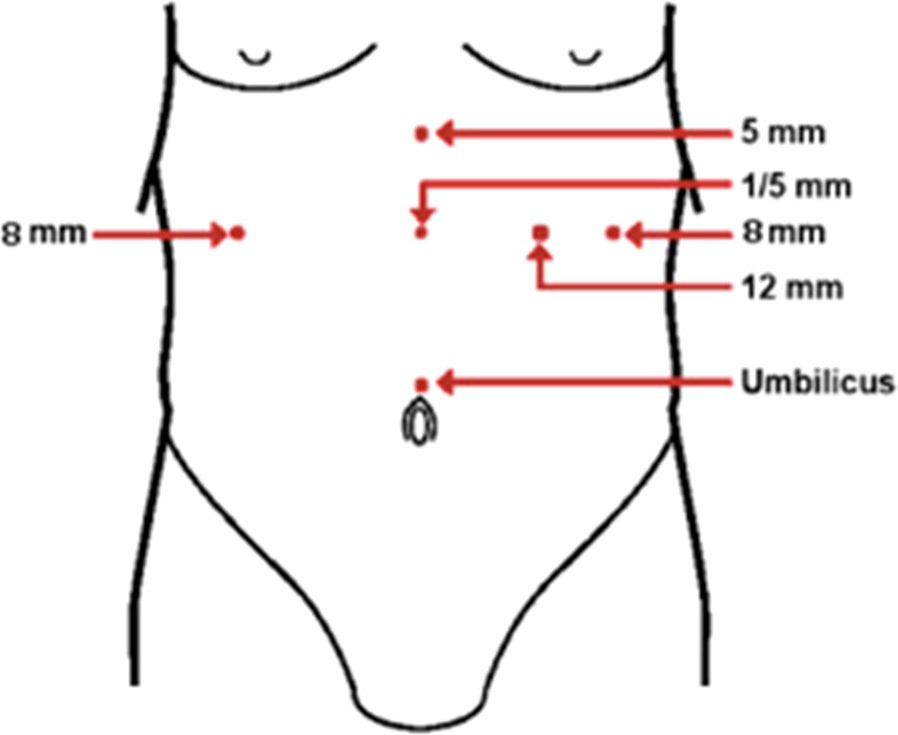
Surgical team disposition in laparoscopic adjustable gastric banding. (Moser and Horgan [15])

The patient must be placed in the low lithotomy position with the legs and arms open. The surgeon operates between the patient’s legs, with the assistant at the patient’s left side (Fig. 2).

**Fig. 3:**
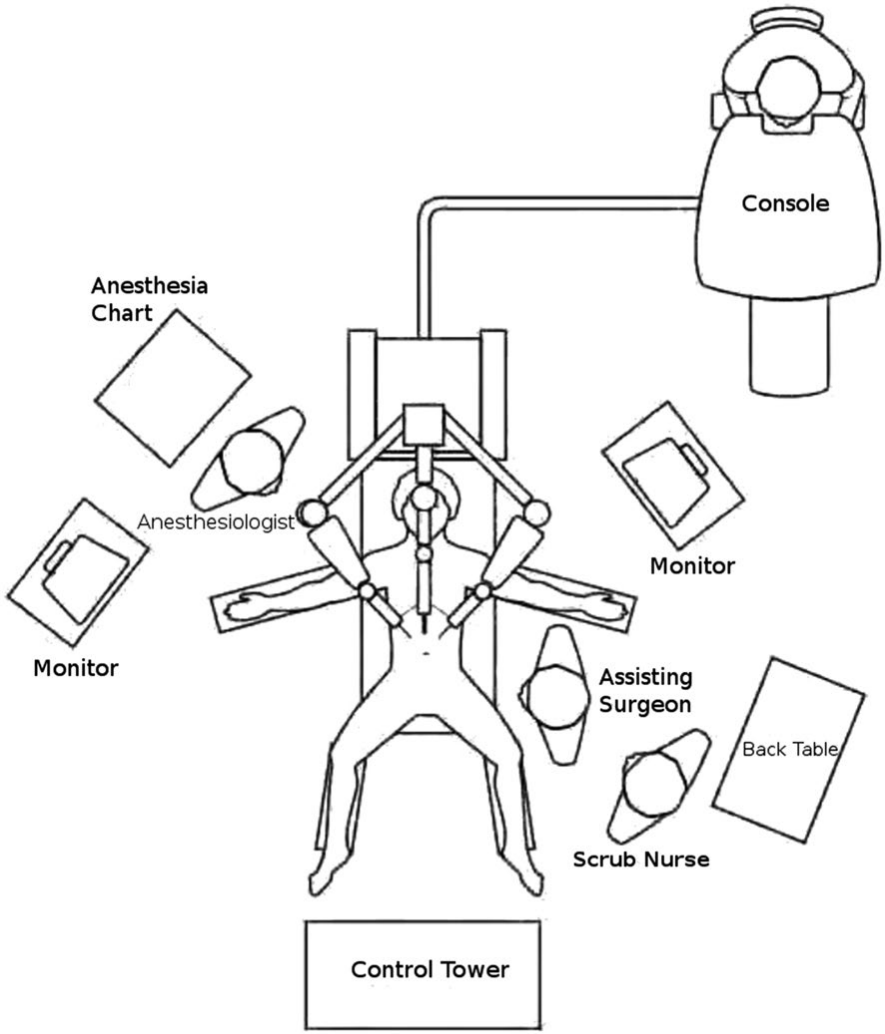
Illustration of trocar placement for robotic and laparoscopic. (Edelson et al. [13]). Adjustable gastric band placement

The first trocar used is a 10- to 12-mm trocar, which is inserted under direct vision or with an optiview trocar, 15–20 cm from the xyphoid process using a 10-mm, 0 or 30° scope, the rest of the trocars are introduced under direct vision. An 8-mm trocar (robotic arm) is placed immediately below the left rib cage in the mid clavicular line; also a 12-mm trocar is then placed on the left flank at the same level as the camera. Then, the patient is placed in the reverse Trendelenburg position, to allow a better visualization of the His Angle. A liver retractor is inserted through a 5-mm incision placed below the xyphoid process. The last 8-mm trocar (robotic arm) is placed approximately 8 cm below the right rib cage (Fig. 3) [13, 15].

A robotic grasper is used attached to the right arm and the harmonic scalpel to the left arm. The first step should be detaching the phrenogastric ligament to expose the left crura. Once this is done, the gastrohepatic ligament is opened to expose the caudate lobe of the liver, the inferior vena cava and the right crura.

A retrogastric tunnel is created between the edge of the right crura and the posterior wall of the stomach until the articulated tip of the robotic instrument is visualized at the angle of His.

The band is placed inside the abdomen, through the 12-mm trocar and the tip of the tubing is placed between the jaws of the robotic grasper, attached to the left arm, and the band is threaded around the stomach [15].

The tip of the tubing is inserted into the band buckle and locked. After the band is in place, a wrap is fashioned out of the stomach to secure it using several non-absorbable seromuscular sutures. Finally, the port is then secured with non-absorbable sutures or using built-in hooks [15].

### Summary

Due to the low complexity of the laparoscopic gastric banding, the utility of the robot in this procedure is debatable. Although there are not many papers reviewing the robotic approach of the lap band, most series tend to show that the robotic and conventional approaches are similar in complication rates and length of postoperative hospital stay but the operating time tends to be longer with the Robot due to the docking.

Probably the most important advantage of the robot can be found when performing a revisional case due to complications or when the lap band must be converted to another bariatric procedure.

## Sleeve gastrectomy

The sleeve gastrectomy (SG) is a restrictive procedure in which a partial left gastrectomy of the fundus and body of the stomach is performed in order to create a long tubular “sleeve” along the lesser curvature. The weight loss and resolution of comorbidities are attributed not only to the restrictive nature of the procedure but also to restriction by the pylorus, decreased ghrelin, increased satiety, increased gastric emptying, and faster small bowel transit times with a component of malabsorption [16–19].

The SG evolved over time from other procedures. In 1988, Doug Hess performed the first sleeve gastrectomy as part the duodenal switch [20]. Anthone in 1997, while performing a duodenal switch in a young patient with common bile duct stones, limited the procedure to only a sleeve gastrectomy due to the complexity of the procedure. In this specific patient, he observed excellent weight loss results with the sleeve alone. Subsequently, between 1997 and 2001, he completed 21 sleeve gastrectomies with similar results [21]. Regan [14] performed the first laparoscopic sleeve gastrectomy (LSG) in very high-BMI patients as a first stage with subsequent laparoscopic gastric bypass Roux-en-Y (LGBYP).

Recently, the American Society for Metabolic and Bariatric Surgery [22] based on several prospective randomized controlled trials and matched cohort studies, recognized the SG as an acceptable primary bariatric procedure and as a first stage for a Roux-en-Y gastric bypass (RYGB) or a duodenal switch (DS). Furthermore, the SG has been found to have a risk/benefit profile somewhere between that of the laparoscopic adjustable band (LAGB) and the RYGB [23–25].

Although complications are rare, they can be very problematic to treat. Gastric leaks following a sleeve gastrectomy can be a very difficult and complex management problem. The average reported leak rate is approximately 2.7 % [26]. For revisional surgery, it can be greater than 10 % [27]. Leaks are caused by local tissue ischemia combined with increased intraluminal pressure of the sleeve. A tight sleeve is a risk factor for a leak, and it is thought that the size of the bougie used is inversely proportional to the rate of leakage [28]. Patients with a distal stricture or a functional obstruction caused by a spiraling staple line are also at a greater risk.

Stricture or stenosis is most common at the incisura angularis. Proper creation of the sleeve with lateral traction and appropriate bougie size when stapling at incisura is key in preventing strictures. Treatment options for stricture can be endoscopic dilatation, seromyotomy, or conversion to a RYGB.

Although the rate of staple-line dehiscence is low in laparoscopic sleeve gastrectomies, these complications are feared and extremely problematic. It is believed that the current limitations of laparoscopic surgery (such as limited range of motion, poor ergonomics, lack of depth perception, and surgeon fatigue) could be risk factors for these rare but serious complications. Thus, the implementation of the da Vinci system could reduce the incidence of this complications.

As in the pre robotic era, the robotic sleeve gastrectomies (RSG) were also first performed as part of robotic biliopancreatic diversion with duodenal switch (RBPDDS) in 2000 [29]. Series of 39 RSG procedures were reported [30], comparing them to standard laparoscopy (LSG) and longer operative times by 21 min in the robotic group were found; this longer operative time was due to the need to suture over the staple line robotically, whereas the laparoscopic groups staple line was not oversewn.

Other authors haven’t seen differences in outcomes in RSG when compared to LSG, except for longer operative times which were due to docking the robot. Docking times of 16 min were long and likely reflected the learning of efficient docking techniques [31].

Another study comparing LSG versus RSG, involving multiple surgeons, where 277 LSG procedures were reviewed against only 40 RSG procedures, showed that operative times were longer with RSG at 113 min versus 91 min for LSG. However, leak rates were higher with standard laparoscopy, showing 1.8 % leaks in the LSG arm and 0 % in the RSG arm. Time differences were likely due to differences in surgeon technique and revealed weaknesses in the retrospective review [31].

More studies comparing techniques obviously need to be done for primary sleeve gastrectomy; current data show no obvious clinical outcome advantages.

The use of the robot in revisional sleeve gastrectomy cases has significant promise. In patients with sleeve gastrectomy and severe reflux due to inadequately treated hiatal hernias, a formal hiatal dissection and posterior crural repair has been performed in three patients with concurrent plication of the dilated upper portion of the sleeve. This technique has shown good resolution of reflux in the early postoperative period. The dissection and reconstruction of anatomy can be easily accomplish in revisional cases with robotic assistance. Robotics has shown also to be beneficial when performing a sleeve gastrectomy after liver transplantation [32] and helpful to perform stricturoplasty of a strictured sleeve [33].

### Surgical technique

**Fig. 4:**
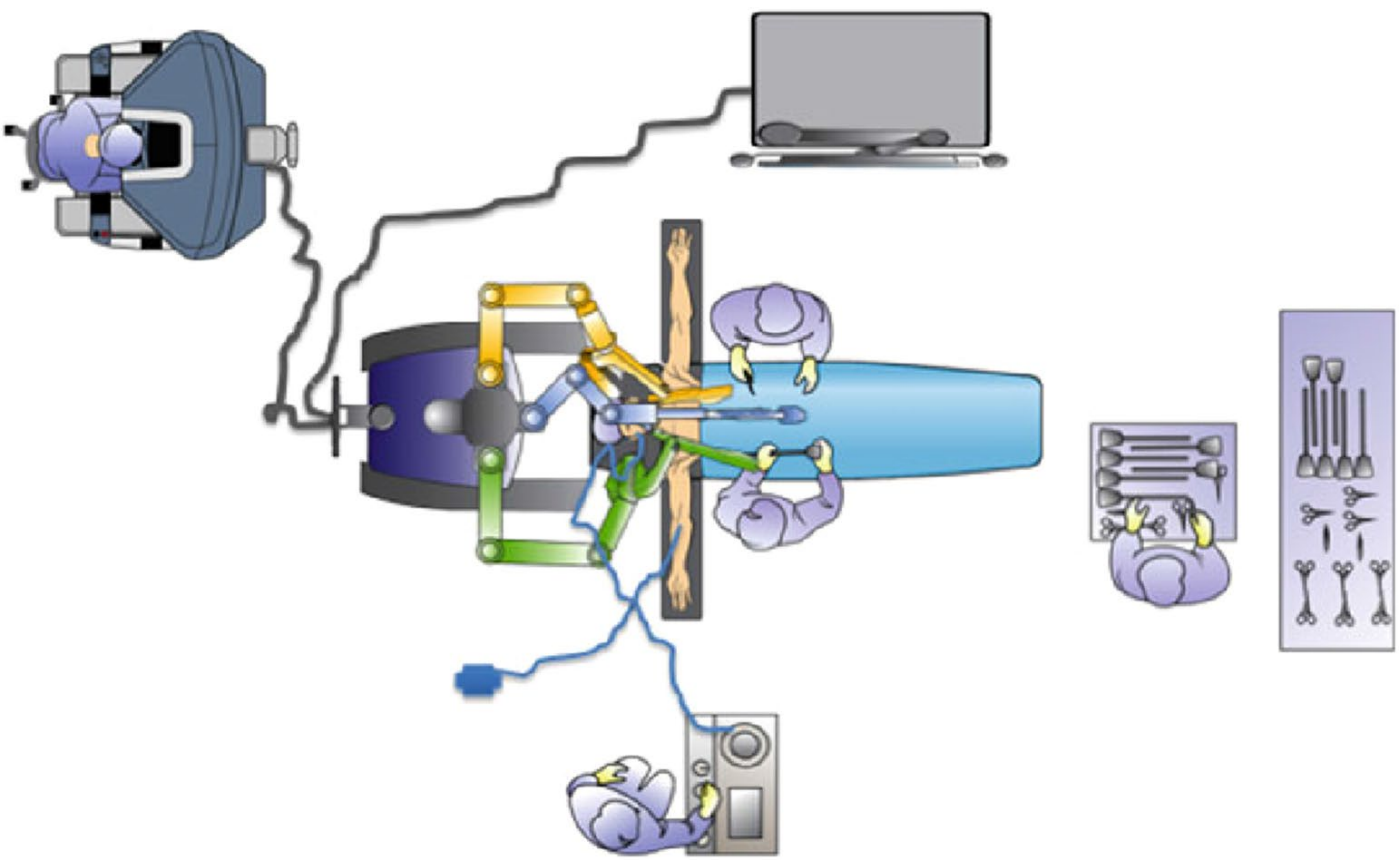
Robotic sleeve gastrectomy OR set up (Rabaza and Gonzalez [49])

The patient is placed in supine position with the arms extended, the robot is docked over the head of the patient, while anesthesia is positioned on the patient’s right side, it is important always to ensure that the anesthesiologist has instant and unobstructed access to the head of the patient (Fig. 4). The bedside assistant stands on the patient’s right side and the robotic monitor is placed across from the assistant on the patient’s left.

The patient should be draped without the anesthetic barrier in order to allow the robot to be docked over the head. Prior to docking the robot, the patient is placed in the reverse Trendelenburg position at 15°–20°.

### Trocar placement

Three trocars for robotic arms plus an assistant trocar are placed. The camera trocar, which is a 12 mm long trocar, is positioned above the umbilicus. The two robotic working arms, which can be 5 or 8 mm robotic trocars, are positioned at the anterior axillary line on both sides and just above the level of the camera port. A 12 mm non-robotic port is then placed approximately halfway between the line from the umbilical port to the right robotic port and slightly inferior. The liver can be retracted with a Nathanson Hook Liver Retractor, which is placed just below the xiphoid and held in place with a retractor that is mounted to the bed over the patient’s right shoulder. Finally the robot is docked directly above the patient’s head.

The pylorus must be identified as a first step. Approximately 4–6 cm proximal to the pylorus, the vascular attachment of the gastrocolic ligament is divided with the use of an energy source such as the Harmonic scalpel.

Once it is decided the area where the dissection is going to begin, the console surgeon grasps the stomach with a bowel grasper and gently elevates it while the assistant provides counter traction of the gastrocolic ligament. To avoid injuries of the underlying colon it is important to stay close to the stomach. Once the lesser sac is entered, the dexterity of the console surgeon’s left grasper allows easier orientation of the Harmonic scalpel along the greater curvature. Another option is to tuck the left grasper under the stomach and elevating it for further exposure.

The dissection continues to cephalic toward the angle of His and the short gastric vessels. Once the short gastric vessels are located, we must be very careful to avoid troublesome bleeding. Here is where the high definition, three dimensional view of the robot provides an important advantage. Another option is to divide the short gastric vessels after completing the gastric stapling portion, which allows the specimen to be retracted laterally and the vessels to be approached medially, which often provides a better and safer exposure for dividing the gastrosplenic attachments and the short gastric vessels.

After the short gastric vessels are divided at the upper pole of the spleen, the attachments between the fundus and left crus must be divided in order to avoid a large fundus at the superior portion of the stomach (neofundus) and to clearly identify the gastroesophageal junction and to avoid stapling close to this area.

The next step should be the dissection in the area of the phrenoesophageal ligament in search of an occult hiatal hernia. If a hernia is identified, it should be repaired in order to avoid GERD later on. The dissection in this area will also help identify the GE junction in the finals steps of the SG.

Then the distal portion the gastrocolic ligament can be divided to approximately 4–6 cm proximal to the pylorus. Once this is completed, the usually flimsy posterior adhesions of the stomach to the underlying pancreas are divided in order to fully mobilize the stomach. Mobilization is not complete until the lesser curvature vessels are identified from the posterior aspect of the stomach. This will avoid a larger than intended sleeve construction.

Once the vessels are divided and the stomach is well mobilized, the creation of the gastric sleeve begins. First the anesthesiologist has to remove every orogastric tube or probe and pass carefully the 32–36 Fr orogastric bougie which will be used to calibrate the gastric pouch. The bedside assistant surgeon provides lateral traction of the stomach, while the console surgeon, with the aid of the articulating bowel grasper, guides the bougie into the proximal duodenum.

Once the calibration bougie is in place, the transection begins. It is important to pay attention to the angle of the stapler and its proximity to the incisura angularis. Because of the tissue thickness in this area, the first firing should be performed with a green cartridge of 60 mm stapler (2.0 mm). The console surgeon again retracts the tip of the bougie medially toward the duodenum with the articulating left-hand grasper and lateral retraction of the greater curvature with the right hand. The assistant bedside surgeon then introduces the stapler. The stapler is placed across the antrum in a more horizontal than vertical orientation. This technique allows a “wide turn” at the area of the incisura, obviating a stricture or spiraling.

The transection continue proximally along the lateral edge of the bougie while maintaining lateral symmetrical traction. This is important to avoid letting the staple line to spiral either anteriorly or posteriorly because this can lead to a functional obstruction. This step is greatly facilitated by the dexterity and maneuverability of the robotic wristed instruments. This portion of the transection due to the tissue thickness, can be performed with a blue cartridge (3.5 mm).

The final critical step is the completion of the transection at the angle of His. Most bariatric surgeons generally stay away from the gastroesophageal junction during the last staple firing in order to avoid a leak in this area which can be catastrophic. However, leaving too large a fundus can also be a problem because it can lead to insufficient weight loss or incapacitating gastroesophageal reflux.

After completing the sleeve, many surgeons reinforce the staple line in order to decrease the incidence of bleeding and leaking [34], this maneuver can be performed much more easily with the help of the robot. If an imbricating suture is used, then it should be done with the bougie in place.

Once the procedure is completed, the staple line is carefully examined for bleeding and for spiraling. If spiraling is found, the previous divided gastrocolic fat is sutured to the staple line to prevent kinking or further spiraling.

As a final step, an intraoperative endoscopy should be performed in order to ensure an intact staple line with air leak test and an uniform unobstructed lumen.

Generally, a drain is not necessary with most cases, but should be considered in difficult or revisional cases. The resected stomach is removed via the assistant port site or the umbilical site. As always, this fascial site should be closed to prevent an immediate postoperative incarcerated incisional hernia.

### Summary

The use of the robot in bariatric surgery has been restricted only to those surgeries that are considered complex, such as revisions or bypass surgery; there are only a few papers that report the use of the robot for sleeve gastrectomies (Table 1).

**Table 1:** Review of the literature reporting the use of the robot for sleeve gastrectomies

	Diamantis et al. [31]	Ayloo et al. [30]	Abdalla et al. [50]	Elli et al. [32]	Vilallonga et al. [51]	Gonzalez et al. [52]
Year	2011	2011	2012	2012	2012	2012
Number of patients	19	30	5	1	32	134
Leaks	0	0	0	0	0	0
Strictures	0	1 (3.3 %)	0	0	0	0
Bleeding	0	0	1 (20 %)	0	0	1 (0.7 %)
Mortality	0	0	0	0	0	0
Conversion	0	0	NP	0	0	0
Surgical time	95.5 ± 11.5	135 ± 28		158	77.5 (56–130)	106.6 ± 48.8
Hospital length of stay	4	NP	NP	4	NP	2.2 ± 0.6

Comparing the three most common major complications after an leak, bleeding, and stricture (LSG) as well as the surgical time and hospital length of stay, both laparoscopic and robotic techniques are safe and feasible, showing good results in every measured parameter. However, surgical time tend to be faster during the laparoscopic approach, and hospital length of stay tend to be shorter with the robotic approach.

As a weak point of the RSG we can highlight the lack of a robotic stapler, which essentially assigns the stapling portion of the procedure (the most critical portion of the procedure), to the bedside surgeon. But in the other hand, the enhanced dexterity of the robot greatly facilitates reinforcing the staple line by suturing.

## Gastric bypass

The gastric bypass procedure was developed in the 1960s by Dr. Mason [35] and based on the weight loss observed after ulcer treatment in which patients had part of the stomach removed. Over the decades the procedure has been modified into the current form using a Roux-en-Y limb of intestine to produce the Roux-en-Y gastric bypass (RYGBP).

**Fig. 5:**
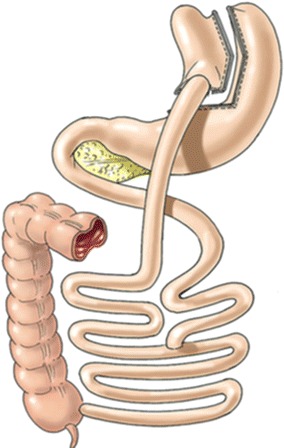
Roux en Y gastric bypass

The Roux-en-Y connects a limb of the intestine to a much smaller stomach pouch which prevents the bile from entering the upper part of the stomach and esophagus, thereby effectively bypassing the remaining stomach and first segment of the small intestine (Fig. 5).

In 1994, Wittgrove et al. reported the first gastric bypass performed via a laparoscopic approach [36]. Since that report, the laparoscopic approach has been adopted widely. With experience using the laparoscopic approach and additional advancements in the field of bariatric surgery, the morbidity and mortality of this operation have decreased to the present very low levels. But in the other hand, unfortunately the laparoscopic approach also introduced significant postural stresses on the surgeon due to the body habitus of the patient. The advent of robotic-assisted Roux-en-Y gastric bypass (RARYGB) eliminated the stresses on the surgeon and introduced several additional enhancements [37]. Minimally invasive surgeons who adopted robotic digital platforms early on have developed refinement of techniques and protocols that lead to safe and effective applications for Roux-en-Y gastric bypass with very low reported morbidity and mortality [38].

Studies comparing the complication rates of the robotic approach against the standard laparoscopic techniques shows lower morbidity and mortality rates for robotic procedures [39]. Also the surgeon’s learning curve during the first 100 robotic gastric bypasses has been reviewed and no anastomotic leaks or mortality were found [40].

Standard laparoscopic gastrointestinal leak rates are usually reported up to 6.3 % and mortality up to 2 % [41, 42]. A series of studies between 2002 and 2008 presented data on operative times and complications after robotically assisted Roux-en-Y gastric bypass [37, 40, 42–45]. A total of 603 patients received either totally robotic (129 patients) or a hybrid robotic procedure (474 patients). An average operative time of 201 min was long; however, the leak rate was significantly low at 0.3 % (2 fistulas or leaks). The safety of the robotic operation was supported with a 0 % 30 day mortality.

At the time, the hybrid procedure, consisting of robotic gastrojejunostomy and laparoscopy for the remainder of the case, was more popular. But since 2008, the totally robotic approach has become more common with improved instruments and techniques where the robot is docked at the beginning of the case and the console surgeon performs the entire procedure with the help of a bedside assistant to deploy any staplers needed for creations of the gastric pouch and intestinal reconstruction [38].

Although the operative time tend to be longer with the robotic approach, there are reports of reduced operative times once the learning curve is overcome. For example Sanchez et al. recounted a randomized trial of RARYGB versus laparoscopic RYGB with significantly shorter operative times for the robotic approach. The RARYGB took 130.8 min versus 149.4 min for the LRYGB (p = 0.02). The largest difference was in patients with a BMI >43 kg/m^2^, for whom the difference in procedure time was 29.6 min faster for RARYGB (p = 0.009) [46].

The advantages of the robotic versus laparoscopic hand-sewn gastrojejunostomies have been also studied. Snyder et al. reported a non-randomized cohort study of 356 LRYGB cases against 249 RARYGB which directly compared laparoscopic handsewn versus robotic hand-sewn gastrojejunostomies. Mortality was non-existent in both groups, and major complication rates were similar between the two groups. The gastrointestinal leak rate was significantly lower in the robotic group (p = 0.04): 1.7 % for LRYGB versus 0 % for RARYGB, this emphasize the clinical benefit from the precision of the robot [47].

Among the benefits of the robotic approach we can highlight the advantages that directly benefit the surgeon like a relief from painful ergonomic positioning and postures that affect the neck, shoulders, and back. Also the superior upper abdominal visualization allows for robotic preciseness and allows face the challenge that come from patients with prior abdominal surgeries.

In the morbid obese patient with large thick abdominal walls and large livers due to fatty infiltration, robotics allows for more precise reconstruction of the anatomy and effectively working in small spaces than laparoscopy.

In the other hand, some authors have emphasized the disadvantages regarding the robotic approach related with the steep learning curve for manipulating the robot, needing between 12 and 15 cases to normalize outcomes, extended time to dock the robot, difficult mobilization between quadrants, and lack of tactile sense [40, 45].

Learning a new technology and skills always takes time; however, surveys of robotic general surgeons show the learning curve is related primarily to the setup and docking of the system and this improves with training. Performing Roux-en-Y gastric bypass at a console requires the surgeon to follow the same principles and knowledge based on open and laparoscopic surgery. Having this in mind, it is important that surgeons who are new to robotics first pay attention to proper patient selection, initially screening out patients with BMIs ≥40 until a proficient skill level is achieved. Additionally, a hybrid approach should be use at the beginning to perform the different steps of a gastric bypass until adequate skills are developed to perform the bypass totally robotically [46–48].

In the hybrid approach the robot is docked for a smaller portion of the case and as more experience is added, the robot is utilized for a greater portion of the procedure until total robotic bypass is achieved.

Another approach suggests that early on, many surgeons are best suited to dock only 3 arms of the system until the potential trocar and arm interference issues are understood and managed. The forth arm may be added after the procedure has been tried and analyzed. In the end, robotic surgeons need to evolve their procedures because a standard robotic approach does not usually exist [38].

### Surgical technique

Roux-en-Y gastric bypass for morbid obesity is ranked in the top three most challenging advanced minimally invasive procedures in modern general surgery [38]. As such, many technique variations exists enveloped and an important discussion has revolved around creating the gastric pouch, gastrojejunal anastomosis, and jejunojejunal anastomosis.

The parallel-docking position with the patient’s right arm extended allows better access for anesthesia while leaving the head access open for intraoperative endoscopy and a leak test, performed at the end of the procedure.

Prior to docking the robotic arms, a footboard is positioned and 20° reverse Trendelenburg is used. Finally, a gastric lavage tube is placed preoperatively to facilitate pouch creation and to stent the gastrojejunal anastomosis while sewing.

**Fig. 6:**
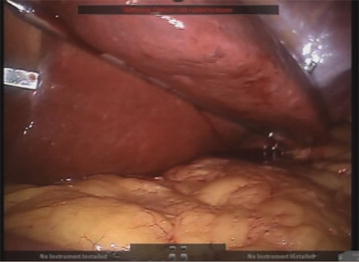
Endolift liver retractor to avoid the epigastric incision

A total of five or six trocar ports are placed for robotic-assisted RYGBP:

1. A peritoneal entry is placed with a zero degree scope on a 5 mm optical viewing in the right upper quadrant just to the right of the midclavicular line, one finger width below the costal margin. This port is subsequently changed to the robotic “number two arm” after all other ports have been placed.
2. A 12 mm umbilical port for the robotic camera,
3. A 5 mm left upper quadrant port placed at the level of the umbilicus at the anterior axillary line with the “number three robotic arm” docked,
4. The area between the umbilical port and left anterior axillary line port is bisected and an 8 mm robotic port is placed with the “number one robotic arm” docked,
5. A 12 mm right mid- abdominal assistant port is placed halfway between the umbilical port and the right upper quadrant port.
6. If the liver is small, a 3 mm retractor or an internal liver retractor (EndoLift™ Port-Free Retractor, Fig. 6) can be used, reducing the need for an epigastric incision. A sixth port is created if the liver is large, in which case an epigastric incision is made to facilitate a Nathanson liver retractor in order to elevate the left lateral lobe.

This trocar placement allows for the RARYGB to be accomplished without the reported inconvenient of moving the robot from one quadrant to another. Both upper and lower quadrants are easily visible and manageable for work without re-placing trocars and extending surgical and anesthesia time.

If we divide the entire procedure into 3 steps, the first step would be the creation of the Gastric Pouch. First, the angle of His is identified with the fundus retracted laterally. The peritoneum, over the angle of His, is dissected with ultrasonic shears or scissors and carried posterior to identify the path for a linear stapler and the left crus of the diaphragm. Next, the pars flaccida is identified and opened. At this point it is important to identify the left gastric artery and its branches onto the lesser curve for preservation, as this will be the main blood supply to the gastric pouch and the gastrojejunal anastomosis. The mesentery to the lesser curve of the stomach is divided by a vascular load linear stapler. A retrogastric plane in the lesser curve is then created and the dissection is carried up to the angle of His. Once accomplished, two serial applications of a 60 mm linear stapler are used to create a 20 mL gastric pouch.

The second step would be the creation of the Jejunojejunostomy. The jejunum is measured and, a distance of approximately 50–70 cm of the ligament of Treitz must be divided with a linear stapler with a white load. Then through the use of the robotic hook, the mesentery is sectioned to gain limb mobility. By using the two robotic graspers, the distal limb of the divided jejunum (the Roux limb) is measured until 150 cm and draped into the right upper quadrant. At this point the number three robotic arm is utilized to place a stay suture at the estimated distal staple line and line up the bowel with the direction of the linear stapler. The robotic hook is then used to make the enterotomies, followed by a 60 mm linear stapler to create the anastomosis. The common enterotomy that remains is closed with a single running layer of 2-0 Vicryl. After the creation of the jejunojejunostomy, a silk suture is used to close the mesenteric defect between the Roux limb and the biliary limb of the small bowel.

Finally, the Gastrojejunal anastomosis must be created. The greater omentum is divided with an ultrasonic scalpel, to the level of the transverse colon in order to avoid traction over the Roux limb and de gastrojejunal anastomosis. The Roux limb is pulled up to the level of the gastric pouch.

Once the area to be anastomosed has been identified, the number three robotic arm is used to maintain and properly orient the jejunum in the upper abdomen. The outer posterior layer of the anastomosis is created first using a long 2-0 Vicryl suture.

After the posterior outer layer is completed, the suture and needle are left in situ and attention is focused on constructing the inner layer of the gastrojejunal anastomosis. Using the number two robotic arm, the gastrotomy and enterotomy are performed with the robotic hook.

The inner layer of the anastomosis is also performed with a running 2-0 Vicryl suture. Once the bowel has been opened, the posterior inner row is created. After this step has been performed, the gastric tube placed preoperatively is advanced under guidance of the operating surgeon into the jejunum and facilitates sewing the remainder of the gastrojejunostomy. Once the inner layer is completed, the anterior outer layer is constructed with the same running suture from the posterior outer layer that was left in situ. It is typical that the outer and inner layers are both done with a continuous running suture.

At this point, an intraoperative endoscopy is performed to evaluate a gastrojejunostomy. This ensures passage of the gastroscope into the Roux limb and ensures passage is airtight. The robot is then undocked.

### Summary

Although more studies are necessary, RARYGB seems to be safe and effective procedure and may reduce the learning curve of gastric bypass. Although the operative time might be increased initially, the complication rates, most notably of anastomotic leak, are extremely low. The cost of the procedure is still one of the main concerns. But if we reduce the complication rates by using the robotic approach maybe the cost is not as high as it would seem (fewer reoperations and shorter length of hospital stay) and as more industry investments continue and more competition develops in this area the cost will go down.

## Conclusions

Since Dr. Cadiere and Himpens in the year 1999 performed the first robotic bariatric procedure (a robotic-assisted adjustable gastric banding), the introduction of robotics in the bariatric field has evolved steadily. Sure there is yet a long way to go, and issues like the elevated cost and the prolonged surgical time are problems to solve. And probably as more industry investments continue and the competition among companies grows in this area the cost will go down.

Placing a digital platform between the surgeon and the patient may be scary at first, but in fact it allows for complete measurement of every move the surgeon makes. This could result in more precise evaluations, based on objective data. The digital platform also allows enhancing what the surgeon sees thanks to the 3D vision of the robot. Haptic sensation, which is a common weakness for laparoscopy and robotics, could be magnified so that a sense of ‘‘touch’’ could be better than the human hand.

The main advantages of the robotic system applied to the gastric bypass appear to be better control of stoma size, avoidance of stapler costs, elimination of the potential for oropharyngeal and esophageal trauma, and a potential decrease in wound infection. While in the sleeve gastrectomy and adjustable gastric banding its utility is more debatable, giving a bigger advantage during surgery on patients with a very large BMI or revisional cases.

More studies and data will be needed, but there is a high chance that robotics will ultimately become widely adopted as surgeons learn these new platforms and procedures.

Costs, marketing, and politics can all affect the adoption rates of new technology but, they cannot stop technology that is truly enabling. Robotic digital platforms represent such a technology. The future of digital platforms is robust and the current robotic platforms are just the first steps toward an entirely precise form of bariatric surgery.
